# Raman Studies on Surface-Imprinted Polymers to Distinguish the Polymer Surface, Imprints, and Different Bacteria

**DOI:** 10.1021/acsabm.1c01020

**Published:** 2021-12-23

**Authors:** Birgit Bräuer, Felix Thier, Marius Bittermann, Dieter Baurecht, Peter A. Lieberzeit

**Affiliations:** University of Vienna, Faculty for Chemistry, Department of Physical Chemistry, Waehringer Strasse 42, 1090 Vienna, Austria

**Keywords:** molecularly imprinted polymers, Raman microscopy, partial least squares discriminant analysis, *Escherichia coli*

## Abstract

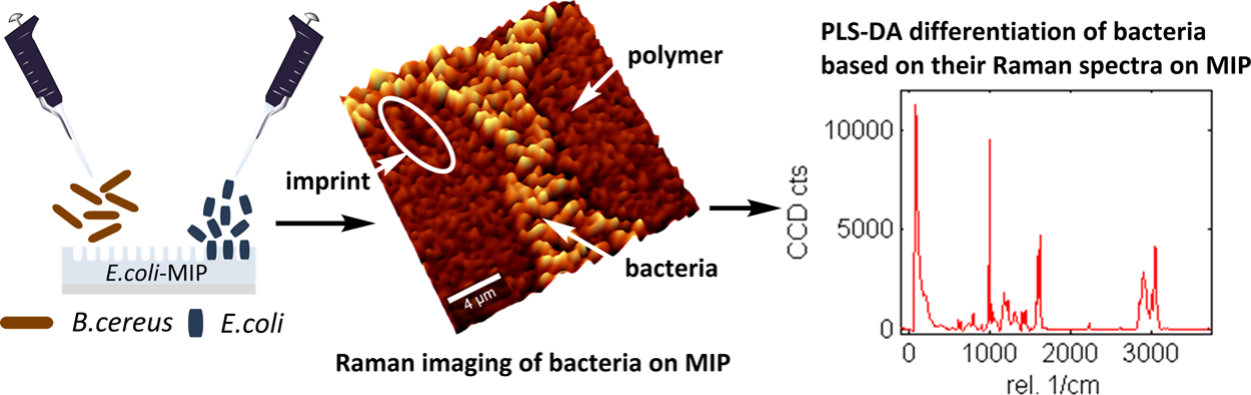

Molecularly imprinted polymers (MIPs) are widely used as robust biomimetic recognition layers in sensing devices targeting a wide variety of analytes including microorganisms such as bacteria. Assessment of imprinting success and selectivity toward the target is of great importance in MIP quality control. We generated *Escherichia coli*-imprinted poly(styrene-*co*-DVB) as a model system for bacteria-imprinted polymers via surface imprinting using a glass stamp with covalently immobilized *E. coli*. Confocal Raman Microscopy was successfully employed to visualize bacteria, imprints, and polymer and to distinguish them from each other. The method has proven highly feasible for assessing if imprinting had been successful. In addition, we developed a method for selectivity investigation of bacteria MIPs based on combining Confocal Raman Microscopy and Partial Least Squares Discriminant Analysis (PLS-DA). The Raman spectra of *E. coli* and *Bacillus cereus* were acquired on *E. coli*-imprinted poly(styrene-*co*-DVB) and used to establish a PLS-DA model for differentiating between the bacteria species. Model validation demonstrated a correct classification of 95% of Raman spectra, indicating sufficient accuracy of the model for future use in MIP selectivity studies. Simultaneous differentiation of 3 bacteria species (*E. coli*, *B. cereus*, and *Lactococcus lactis*) on *E. coli*-imprinted poly(styrene-*co*-DVB) proved more difficult, which might be due to the limited depth resolution of the confocal Raman microscope resulting in the presence of interfering signals from the polymer substrate. It might be possible to overcome this obstacle by selective enhancement of the Raman signals originating from bacteria surfaces, such as tip enhanced Raman spectroscopy.

## Introduction

It is important to detect and identify pathogenic bacteria not only in clinical environments, where multidrug-resistant bacteria are becoming a serious threat to public health,^1^ but also in food and water safety. Especially, *Escherichia coli* (*E. coli*) is considered a meaningful indicator of food spoilage and environmental hygiene.^2^ Reliable and rapid bacteria detection methods are therefore required to prevent food-borne illnesses. Both conventional techniques, such as plating and culturing, and newer approaches including flow cytometry,^3^ polymerase chain reaction,^4^ and enzyme-linked immunosorbent assay^5^ offer limited sensitivity and selectivity and/or are often time-consuming, require growth and/or enrichment of bacteria, and lack versatility.^6^

To overcome these limitations, *E. coli*-sensitive sensing devices have been developed based on *E. coli*-selective molecularly imprinted polymers (MIPs) as artificial receptors combined with transducers such as quartz crystal microbalances (QCMs) for direct detection of the microorganism in aqueous solution.^7^ The use of MIPs is a widespread approach to fabricate artificial alternatives to natural receptors utilized in the detection of biomolecules. In contrast to natural antibodies,^8,9^ enzymes,^10^ DNA,^11^ or whole cells^12^ used as recognition elements in conventional biosensing, MIPs offer high physical and chemical stability, avoid time-consuming and costly isolation and purification processes, and are readily compatible with a wide range of transducers for signal read-out. These include QCMs,^13^ impedance spectroscopy,^14^ and thermal detection.^15,16^

In MIP fabrication, polymerization takes place in the presence of the template using a mixture of functional monomer(s), a crosslinker, and an initiator. During polymerization, a complex forms between the functional monomers and the target analyte. Extraction of the template following complete curing of the polymer yields cavities that are complementary to the analyte not only in shape but also in surface chemistry. Selective recognition of the target by the MIP cavities is assumed to result not only from template shape but also from interactions such as hydrogen bonds or dipolar bonds between the template surface and functional groups in the polymer matrix.^17^

Assessment of imprinting success and selectivity as well as analyte rebinding studies is of utmost importance to characterize MIPs that are used as recognition layers in sensing devices. Rebinding assessment of bacteria-imprinted polymers is important as there are some challenges associated with imprinting of bacterial cells. They are not uniform in size but rather show a certain size distribution.^18^ For efficient rebinding, the bacteria in the solution to be analyzed should ideally exhibit the same size distribution as the cavities. It has been reported for yeast cells^19^ that rebinding behavior indeed depends on cell sizes used for imprinting.

Herein, we used *E. coli*-imprinted poly(styrene-*co*-DVB) as a model system to develop an approach using Confocal Raman Microscopy to investigate imprinting efficiency, analyte rebinding, and MIP selectivity. Integrating Raman spectroscopy with MIPs has been reported previously in numerous publications,^20−31^ most of which utilize Surface Enhanced Raman Spectroscopy (SERS) to detect the target analyte, resulting in highly selective and sensitive sensors for a variety of target analytes comprising mainly small molecules.^20−30^ Very few papers focusing on Confocal Raman Microscopy as a detection method in combination with MIPs are available.^31^ To the best of our knowledge, no report of utilizing Confocal Raman Microscopy as a technique for MIP selectivity studies has been made to date. Our approach relies on differentiating distinct bacteria strains on the *E. coli*-MIP based on their respective Raman spectra. Thus, the approach is designed to allow for assessing selectivity directly from different bacteria species in a mixture competing for binding to the imprints and thus in one step in situ. In contrast, MIP selectivity tests using, e.g., QCMs^32^ require separate measurements for each species.

However, the concept presented herein requires the ability to distinguish between the polymer, bacteria imprints in the polymer, and especially between different bacteria species on the MIP. One cannot achieve this in a reliable manner using optical microscopy: it lacks the possibility to obtain information on vertical sample topography and chemical composition. Using Confocal Raman Microscopy, a combination of Raman spectroscopy and confocal microscopy, combined with atomic force microscopy (AFM) fulfills all requirements needed. A confocal Raman microscope acquires single Raman spectra at chosen individual positions or multiple spectra within selected areas (2D) or volumes (3D).^33^ Raman spectra contain information on the molecular characteristics and structure of the sample within the investigated area.^34^ Moreover, the resulting dataset makes it possible to generate false-color images showing the spatial distribution of Raman signals corresponding to distinct surface chemistries. It thus indicates areas comprising polymer, imprints, and different bacteria.

However, differentiating between very similar Raman spectra of distinct bacteria species is a very complex task. This is not possible by “simple” analysis of single band intensities. Thus, one needs to apply multivariate data analysis, such as Partial Least Squares Discriminant Analysis (PLS-DA).^35^ Multivariate data analysis is a powerful tool that has been used previously in combination with MIPs to identify and quantify compounds mostly when using spectroscopic methods, such as SERS,^22^ fluorescence,^36^ or microwave spectroscopy,^37^ but also other techniques including square wave voltammetry^38^ for detecting the analyte. It is known that using chemometric tools such as PLS-DA to distinguish bacteria based on Raman spectra in general is feasible.^39−42^ However, acquiring the Raman spectra of bacteria on polymer substrates leads to a substantial number of additional bands stemming from the substrate. It is impossible to avoid such Raman signals of the polymer environment because of the given spatial resolution of the instrument. However, those Raman signals can vary due to inhomogeneities in the polymer structure and additionally may be much more intense than the signals originating from the bacteria. Thus, differentiating bacteria during MIP selectivity studies leads to a much more complex data analysis. Nevertheless, combining Confocal Raman Microscopy and PLS-DA allowed us to successfully develop a model to differentiate two distinct bacteria strains (*E. coli* and *Bacillus cereus*) on a polymer surface, which provides a new approach to investigate the selectivity of polymer surfaces imprinted with bacteria.

## Experimental Section

### Materials and Reagents

*E. coli* ATCC 9637, *L. lactis* ATCC 11454, and *B. cereus* ATCC 11778 were purchased from the American Type Culture Collection (ATCC). *E. coli* BL21 (DE3) and *E. coli* XL1 were obtained from the lab of Prof. Christian Becker at the University of Vienna and used without further cultivation. Microscope slides were obtained from VWR.

d-Glucose monohydrate, (3-aminopropyl)triethoxysilane (APTES), di-potassium hydrogen phosphate, potassium dihydrogen phosphate, dimethyl sulfoxide (DMSO), divinylbenzene (DVB), and styrene were supplied by Merck. Proteose peptone was obtained from VWR chemicals. Yeast extract, 2,2′-azobis(2-methylpropionitrile) (AIBN), and toluene were purchased from Sigma Aldrich. NaCl was supplied by AppliChem. [3-(Methacryloyloxy)propyl]trimethoxysilane was obtained from Alfa Aesar. All chemicals were used as received without further purification.

### Bacteria Cultivation

*E. coli* ATCC 9637 and *L. lactis* ATCC 11454 bacteria were freshly cultured for 24 h at 37 °C in lysogeny broth containing 10 g/L proteose peptone, 5 g/L NaCl, 5 g/L yeast extract, and 1 g/L d-glucose monohydrate and washed twice under sterile conditions with autoclaved distilled water before use.

*B. cereus* ATCC 11778 were freshly cultured at 30 °C for 24 h in lysogeny broth of the same composition and washed twice with autoclaved distilled water under sterile conditions prior to use.

### Synthesis of *E. coli*-Imprinted Poly(styrene-*co*-divinylbenzene)

**Scheme 1:**
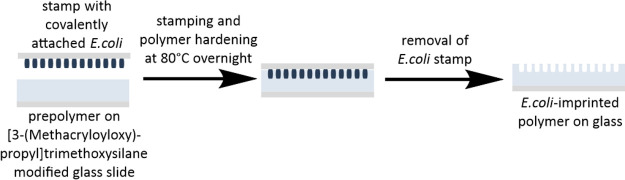
Fabrication of *E. coli*-Imprinted Poly(styrene-*co*-DVB) Using the Stamp Imprinting Approach A glass stamp with covalently attached *E. coli* was pressed into a layer of partly cured poly(styrene-*co*-DVB) on a [3-(methacryloyloxy)propyl]trimethoxysilane-modified glass slide before the polymer was allowed to harden at 80 °C overnight. The stamp was then removed to obtain the *E. coli*-imprinted polymer on the glass.

As stamp imprinting is a well-established straightforward surface imprinting method^16^ and does not require additional washing steps for template removal when the analyte is covalently attached to the stamp, it was our technique of choice for fabricating *E. coli*-imprinted poly(styrene-*co*-DVB) as outlined in Scheme 1.

For stamp fabrication, glass slides of dimensions ∼1.3 × 1.3 cm were cut from microscope slides, washed with tech. acetone, and plasma-cleaned using the Diener Zepto One plasma cleaner at a pressure of 1 × 10^–3^ mbar and a power of 5 W for 10 min. They were subsequently immersed in a solution of 2.4% (v/v) APTES in toluene for 1 h at room temperature before being washed with toluene and dried at 80 °C. The APTES-modified glass slides were then incubated in a solution of 0.5% disuccinimidyl suberate in DMSO for 1 h at room temperature, followed by washing with 25 mM PBS (pH 7) and drying at 37 °C. *E. coli* suspensions were prepared in distilled water at a concentration of 10^8^ cells/mL. Then, the APTES/DSS modified glass slides were incubated with the bacteria for 2 h at room temperature. Excess bacteria were washed off with distilled water, and the stamps were dried at 37 °C prior to imprinting.

The glass slides modified with [3-(methacryloyloxy)propyl]trimethoxysilane were prepared as a base for the *E. coli*-MIP poly(styrene-*co*-DVB) thin films. The glass slides (∼1.3 × 1.3 cm) were cut as before and cleaned in acetone followed by oxidizing the surface (plasma-cleaner) at a pressure of 1 × 10^–3^ mbar and a power of 5 W for 10 min. They were subsequently immersed in a solution of 2% (v/v) [3-(methacryloyloxy)propyl]trimethoxysilane in toluene for 2 h, washed with toluene, acetone, and distilled water, and dried at 80 °C.

Poly(styrene-*co*-divinylbenzene) was prepared using 9 mg of AIBN as an initiator to which 250 μL of each styrene and divinylbenzene was added. The mixture was pre-polymerized at 70 °C until the gelling point was reached. Subsequently, the prepolymer was spin-coated (2000 rpm, 10 s) onto the modified glass slides (see above) before pressing the *E. coli* stamps into the oligomer layer and curing at 80 °C overnight.

### Confocal Raman Microscopy Instrumentation

All Raman single spectra and image scans were acquired on a confocal Raman microscope (alpha 300 RS; WITec Wissenschaftliche Instrumente und Technologie GmbH, Germany) using a diode laser with an excitation wavelength of 532 nm at a laser power of 8 mW. The laser beam was focused onto the sample surface using an EC “Epiplan-Neofluar” DIC lens with 100× magnification and a numerical aperture of 0.9 (Carl Zeiss AG, Germany). An ultra-high throughput Raman spectrometer was used with a diffraction grating (600 gr/mm, BLZ = 500 nm). Raman-scattered light was detected on a thermoelectrically cooled front-illuminated CCD camera.

### General Parameters and Data Processing of Raman Single Spectra and Image Scans

The integration time and number of accumulations of the single spectra as well as the dimensions, points/line, lines/image, and integration time/spectrum for the Raman image scans are given with the corresponding results. WITec Project FIVE software using the shape function with a shape size of 150 pixels served to subtract the background from the images and single spectra. Cosmic rays were removed utilizing the WITec Cosmic Ray detection algorithm with a filter size of 2 pixels and a dynamic factor (sensitivity of the algorithm) of 12. Raman false-color images displaying signal intensity distribution at 2908 cm^–1^ were created using the WITec Control FIVE software with an average (binomial) filter with a filter size of 20 pixels. Light microscopy images were acquired at 100× magnification using the previously mentioned lens on the WITec confocal Raman microscope.

### Atomic Force Microscopy Instrumentation

AFM measurements took place using the AFM function of the WITec alpha 300 RS in AC mode configuration using 285 KHz 42 N/m reflex-coated acoustic AC mode cantilevers (purchased at WITec Wissenschaftliche Instrumente und Technologie GmbH, Germany) at a scan speed of 1 line/s with a scan dimension of 20 × 20 μm and 512 points/line, 512 lines/image.

### Visualization of Bacteria, Polymer, and Imprints on *E. coli*-Imprinted Poly(styrene-*co*-DVB) Using Confocal Raman Microscopy and AFM

Three different *E. coli*-imprinted poly(styrene-*co*-divinylbenzene) samples were fabricated as described above. A drop of 2 μL of an aqueous *E. coli* suspension of 10^8^ cells/mL was applied to the MIP and left to dry at 37 °C. The resulting sample area mimics successful bacteria rebinding to the MIP as it contains bacteria, imprints, and polymer. Raman and AFM imaging took place as described above at several spots on each surface.

### Differentiating between Bacteria Species on *E. coli*-Imprinted Poly(styrene-*co*-DVB)

**Scheme 2:**
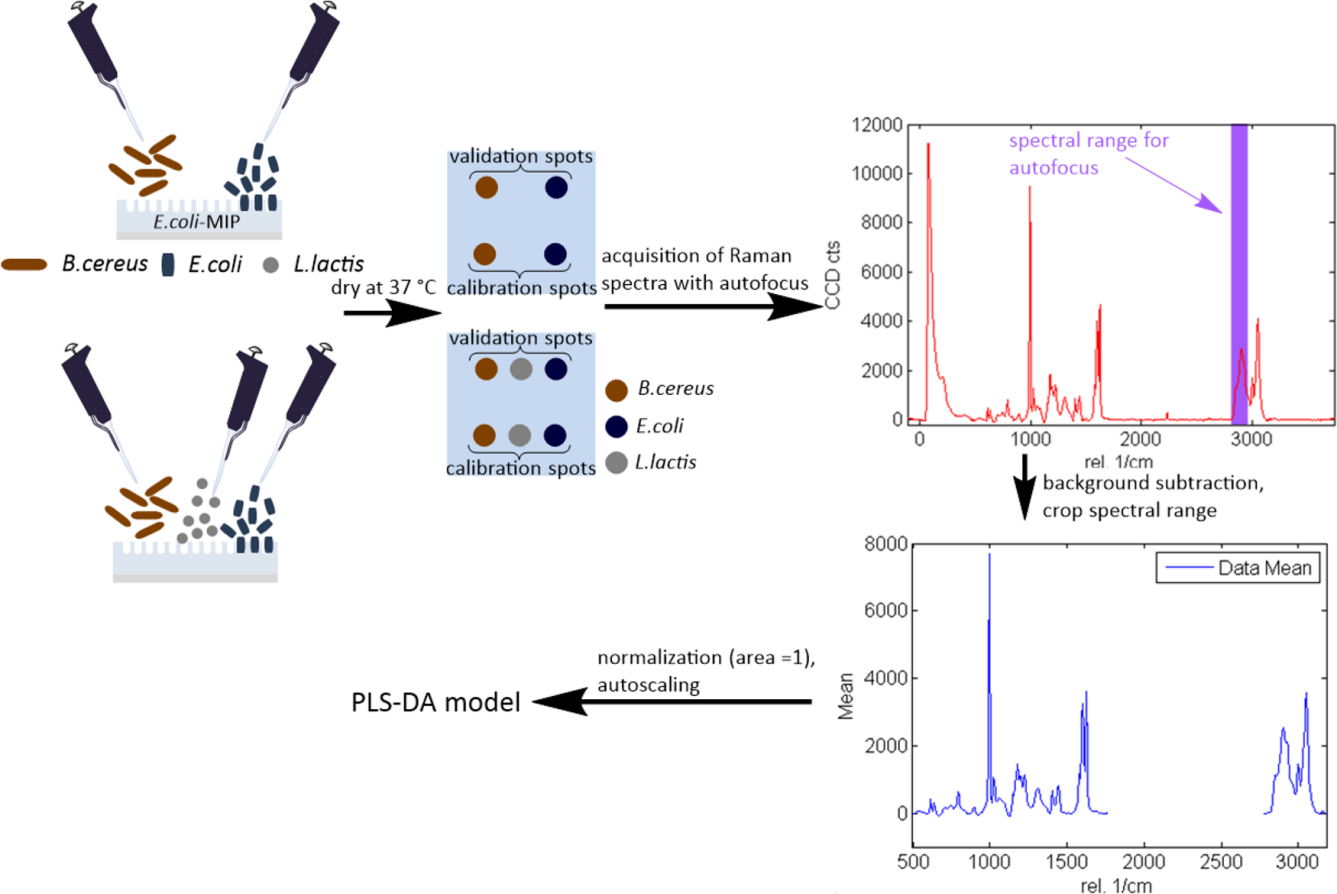
Experimental Setup for Differentiation of Two and Three Bacteria Strains on *E. coli*-Imprinted Poly(styrene-*co*-DVB) via PLS-DA The Raman spectra for each bacteria species were acquired on the *E. coli*-MIP at two separate sample spots treated with the respective bacteria for calibration and validation. Spectral autofocus was performed for maximizing the Raman signal intensity between 2783 and 3008 cm^–1^. The spectra were preprocessed by background subtraction, normalization, and autoscaling, and only bacteria-relevant spectral regions (500–1762 and 2778–3181 cm^–1^) were used for establishing the PLS-DA model.

Suspensions with a concentration of 10^8^ cells/mL were prepared in distilled water for each bacteria strain. Two drops of 2 μL were pipetted onto the *E. coli*-imprinted poly(styrene-*co*-DVB) for each bacteria species and left to dry at 37 °C (see Scheme 2).

Two drops per bacteria strain were used to acquire the calibration (20 spectra per species) and validation spectra (10 per species) to avoid “pseudo-differentiation” of the microorganisms arising from possible differences in local composition of the bulk polymer. Prior to acquisition of each Raman spectrum, spectral autofocus was performed (maximizing the Raman signal intensity in the range of 2783–3008 cm^–1^, which is the wavenumber range where the strongest Raman signal was observed in the “pure” *E. coli* spectrum on CaF_2_). Background subtraction and cosmic ray removal took place as described above. The spectra were imported into SoloMIA chemometrics software (Eigenvector Research Incorporated) for analysis. For each bacteria strain, the same number of spectra from the corresponding calibration spot was used for the PLS-DA model calibration. The spectra were cropped; only the spectral regions containing signals resulting from the bacteria were used for differentiation (500–1762 and 2778–3181 cm^–1^). Subsequently, the cropped data were further preprocessed by normalizing (1-Norm, area = 1) and autoscaling. Preprocessed spectra were assigned their respective class (bacteria species) before the PLS-DA model was calculated. The spectra acquired at the validation spots were preprocessed in the same manner as the calibration dataset. For validation, the PLS-DA model was applied to the test dataset consisting of the same number of spectra for each bacteria strain.

For differentiation between two *E. coli* strains (BL21 (DE3) and XL1), model calibration relied on 20 spectra from two bacteria spots per strain. Applying 20 spectra per strain from those spots to the residual for validation helped compensating for local differences in polymer composition. These are expected to have larger impact on both model calibration and validation, when the bacteria are very similar (which is the case for two *E. coli* strains): the model might not classify bacteria correctly because of the small differences in polymer composition between the calibration and validation spots.

To assess the feasibility of the Raman Microscopy-PLS-DA approach to identify bacteria (namely, *B. cereus* and *Lactococcus lactis*) from a mixture on *E. coli*-imprinted poly(styrene-*co*-DVB), model calibration relied on one calibration spot per bacterium (20 spectra per species). Validation of the model took place using 40 Raman spectra acquired on a mixture of *B. cereus* and *L. lactis* on the *E. coli*-MIP (20 spectra per strain, distinguished by shape). Spectral preprocessing, data preprocessing, PLS-DA model establishment, and validation were carried out in the same manner for all bacteria differentiation experiments.

## Results and Discussion

### Visualization of Bacteria, Polymer, and Imprints on *E. coli*-Imprinted Poly(styrene-*co*-DVB) Using Confocal Raman Microscopy and AFM

**Figure 1:**
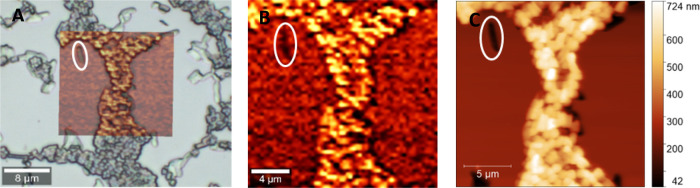
A: Overlay of a Raman image scan of *E. coli* applied to *E. coli*-imprinted poly(styrene-*co*-DVB, simulating bacteria rebinding) (Raman signal intensity distribution at 2908 cm^–1^, shown in B) and the corresponding light microscopy image; B: Raman image scan (20 × 20μm, 60 points/line, 60 lines/image, 0.1 s integration time per spectrum) showing Raman signal intensity distribution at 2908 cm^–1^, C: AFM image (20 × 20 μm, 512 points/line, 512 lines/image) of the same sample area confirming the differentiation between bacteria, imprints, and polymer given by the corresponding Raman image scan (imprint highlighted in white).

In a first step, it is necessary to demonstrate the possibility to distinguish bacteria, polymer, and imprints in the polymer from each other by Confocal Raman Microscopy. Figure 1A shows the overlay of the white light image of an *E. coli*-MIP that is partly occupied (simulating bacteria rebinding to the polymer) with the Raman false color image in Figure 1B. The latter contains the intensity distribution of the Raman signal at 2908 cm^–1^ (Figure 1B). This overlay allows us to reliably distinguish between imprints, non-imprinted poly(styrene-*co*-DVB), and bacteria because they all show distinct signal intensities at 2908 cm^–1^ (aliphatic C–H stretching vibrations; increasing brightness of the color indicates higher corresponding signal intensities): Bacteria lead to high Raman signal intensity (light yellow) at the chosen wavenumber. In contrast, the non-imprinted polymer reveals much lower signal intensity in that spectral region (red color). Finally, imprints lead to the weakest signals at this wavenumber (black) because the laser focus is above the surface in those areas. We confirmed the presence and location of *E. coli*, imprints, and surrounding polymer by the topographic information yielded by AFM of the same sample area (Figure 1C), validating the differentiation suggested by the Raman image. Taking images at different positions of different films confirms these results: in every case, it is possible to distinguish the polymer surface, imprinted areas, and bacteria from each other.

### Influence of Topography on Differentiating *E. coli*-Imprints and Polymer in Poly(styrene-*co*-DVB)

**Figure 2:**
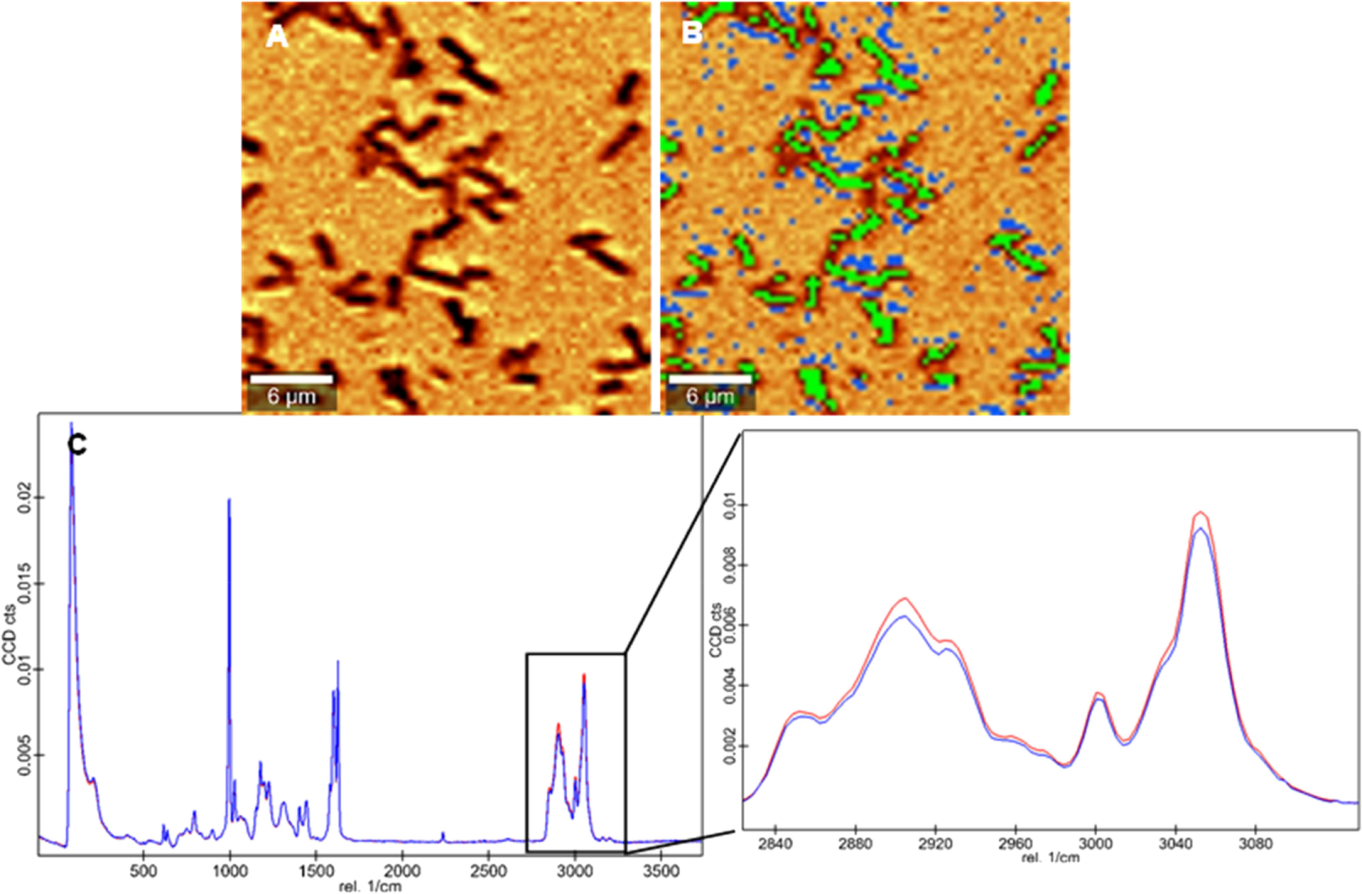
A: Raman image scan (30 × 30 μm, 50 points/line, 50 lines/image, 0.1 s integration time per spectrum; intensity distribution at 2908 cm^–1^), B: green: pixels used for average spectrum of imprints, blue: pixels used for average spectrum of surrounding polymer, C: normalized average spectra of *E. coli*-imprints (blue) and surrounding polymer (red).

Sample topography plays a huge role when differentiating *E. coli*, imprints, and surrounding polymer from each other. Raman signal intensity does not only change when different molecules are present but also when the focus level of the microscope changes its position relative to the sample surface. Therefore, it is important to know to which extent sample topography and surface chemistry contribute to the differences in Raman signals observed when comparing the spectra of imprints and surrounding polymer within the image dataset. Figure 2A shows a Raman image scan of *E. coli*-imprinted poly(styrene-*co*-DVB) after background subtraction and removing cosmic rays for all spectra prior to image analysis. Average spectra were generated (for details, see the Supporting Information) for both *E. coli*-imprints (Figure 2B, green pixels) and surrounding polymer (Figure 2B, blue pixels) to increase the signal-to-noise ratio. At first glance, the average spectrum of the non-imprinted polymer does not differ from the average imprint-spectrum. It appears to exhibit the same Raman signals with increased overall intensity because the laser focus is set to the plane of the polymer surface and not to the plane of the imprint. One can look into this in a bit more detail when normalizing the average spectra of *E. coli*-imprints and surrounding polymer using the 1-Norm (area = 1) function (Figure 2C).

An overlay of the normalized spectra emphasizes that the relative intensities of the Raman signals are visually the same, except for slightly noticeable differences in the signal ranges 2987–3130 and 2820–2983 cm^–1^. This indicates that differentiation of the imprints and surrounding polymer in Raman images largely is the result of topography. However, it does not rule out that differences in surface chemistry between the imprints and non-imprinted polymer also contribute to the differentiation: those could be responsible for very small spectral changes that might only be extracted using chemometric methods. One would expect that the surface chemistries of both imprints and polymer only contribute to the spectra underlying Raman image scans in a minor way given that depth resolution of the confocal Raman microscope is about 800 nm. Hence, a large proportion of the observed Raman signals stems from the bulk of the polymer rather than its surface. Therefore, one needs to enhance the surface signal intensity compared to the bulk polymer to gain deeper insight into the surface chemistry of the imprints and polymer.

### Extraction of the *E. coli* Spectrum from the Spectra Acquired on *E. coli*-Imprinted Poly(styrene-*co*-DVB)

**Figure 3:**
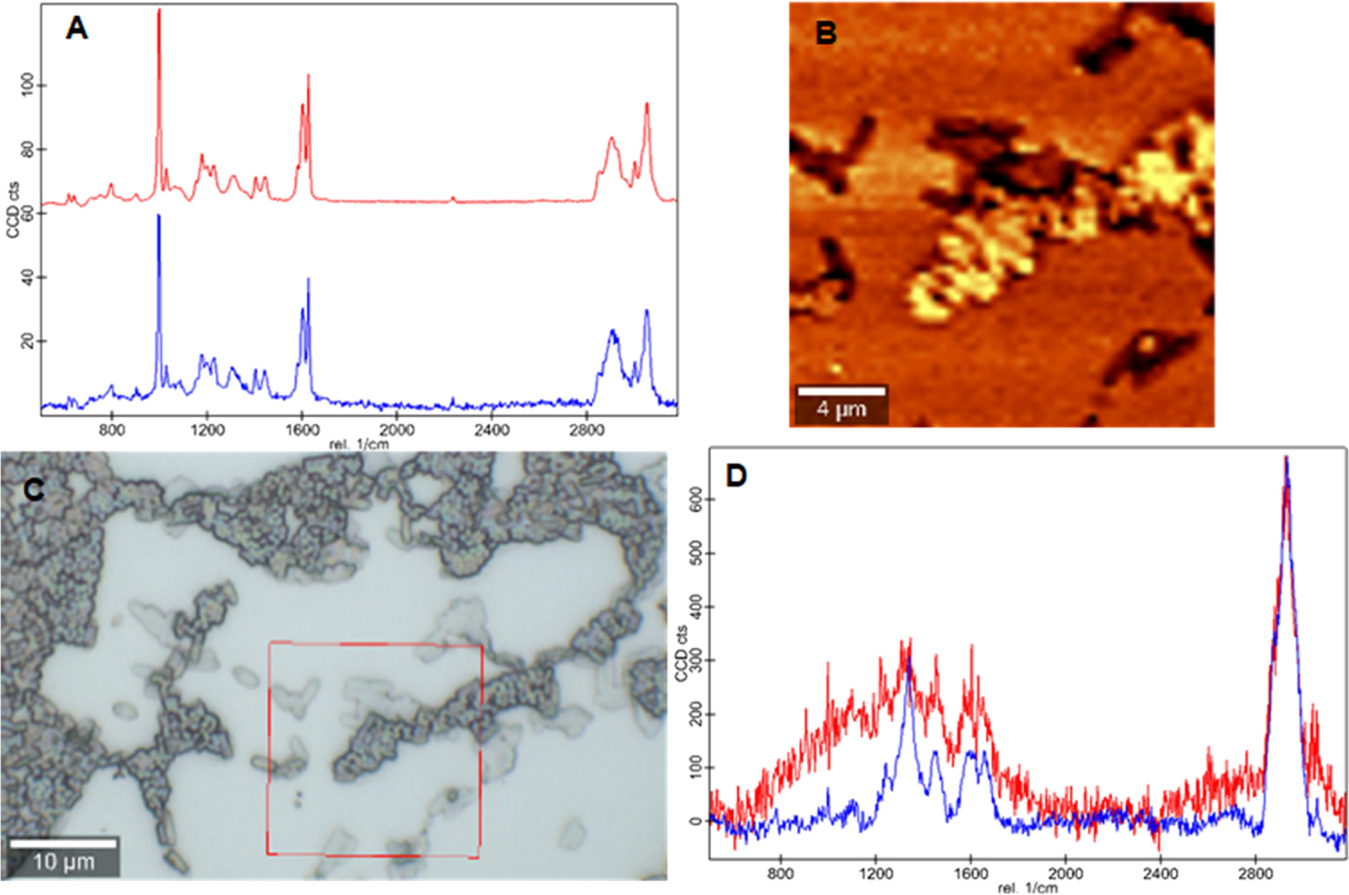
A: Spectra of the two components indicated in C (poly(styrene-*co*-DVB) (red) and *E. coli* located on *E. coli*-imprinted poly(styrene-*co*-DVB) (blue)), B: Raman intensity distribution image at 2908 cm^–1^ of the area indicated in C, C: light microscopy image of *E. coli*-treated *E. coli*-MIP, D: overlay of the residual spectrum after subtraction of component 1 from 2 (red) and spectrum of “pure” *E. coli* acquired on CaF_2_ (blue).

Figure 3A displays the average Raman spectra of poly(styrene-*co*-DVB) (red spectrum) as well as *E. coli* located on *E. coli*-imprinted poly(styrene-*co*-DVB) obtained from the Raman image scan in Figure 3B (blue spectrum, locations indicated in the light microscopy image in Figure 3C). They cannot be distinguished easily from each other: both appear to be pure polymer spectra, which can be attributed to the fact that bacteria are much weaker Raman scatterers than the used polymer. However, de-mixing (for details, see the Supporting Information) of the 2 components in the image scan results in extracting a “pure” *E. coli* spectrum (red spectrum in Figure 3D), which is very similar to a spectrum of *E. coli* on CaF_2_ (blue spectrum in Figure 3D).

This confirms that it is possible to separate Raman scattering arising from bacteria on the polymer from high-intensity substrate signals. Sufficient intensity of bacteria signals on the polymer is a prerequisite to distinguish different microorganisms on imprinted polymers required for selectivity studies based on Raman spectra. However, the poor signal-to-noise ratio of the de-mixed *E. coli* spectrum compared to that of poly(styrene-*co*-DVB) might affect the ability to differentiate bacteria strains on the polymer. In addition, the Raman spectra of individual bacteria strains only differ slightly. This is a result of the fact that bacteria are very similar in overall chemical composition. Furthermore, confocal Raman microscopes have limited depth resolution. Therefore, given the size of bacteria cells, Raman signals originate from the bacterium as a whole and not only from the cell wall.

### Differentiating between *E. coli* and *B. cereus* on *E. coli*-Imprinted Poly(styrene-*co*-DVB)

**Figure 4:**
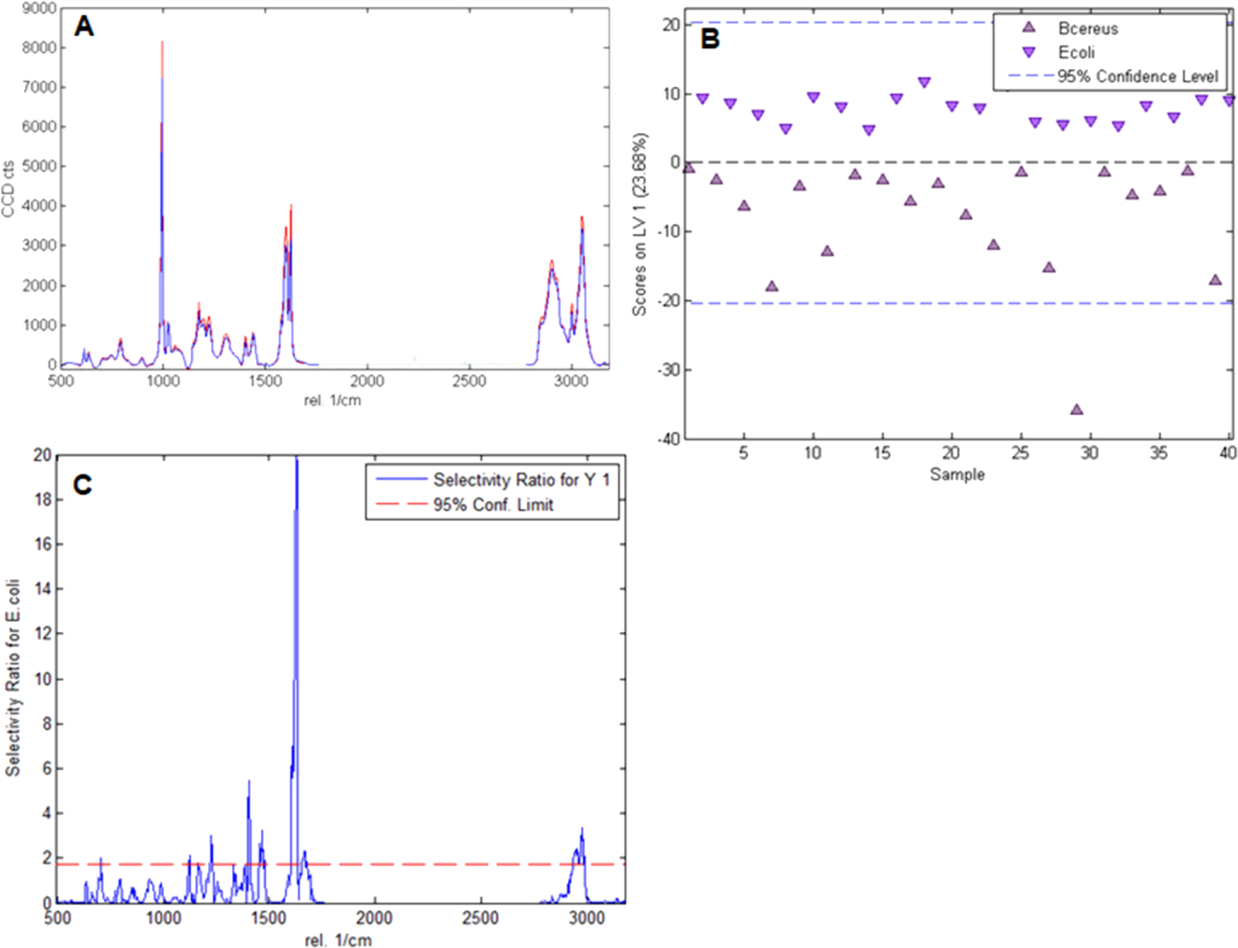
A: Overlay of the mean calibration spectra of *E. coli* (red, average of 20 spectra) and *B. cereus* (blue, average of 20 spectra) on the *E. coli*-MIP showing only the spectral range used for calibration and validation of the PLS-DA model established; B: scores plot of the obtained PLS-DA model showing the first latent variable vs the sample number; C: selectivity ratio indicating the relevant spectral regions for differentiating *E. coli* from *B. cereus.*

Figure 4A, which shows the average spectra for *E. coli* and *B. cereus* on the *E. coli*-MIP (for details on calculation, see the Supporting Information), demonstrates the similarity of the acquired Raman spectra of the bacteria species on the substrate. This necessitates the use of chemometric techniques such as PLS-DA to distinguish the bacteria.

For bacteria differentiation on the MIP, we preprocessed the calibration and validation spectra (20 s integration time and 3 accumulations per spectrum) and established the PLS-DA model as described in the Experimental Section. Figure 4B shows the obtained scores plot from calibrating the PLS-DA model for differentiating *E. coli* and *B. cereus* on the *E. coli*-MIP surfaces. One can clearly observe that the spectra of *E. coli* and *B. cereus* cluster when using a model with only one latent variable: *E. coli* spectra exhibit positive and *B. cereus* spectra negative scores in this case. Figure 4C shows the selectivity ratio for the *E. coli* class, which visualizes signal ranges that are important to distinguish *E. coli* from the *B. cereus* class (signals that extend above the 95% confidence limit are of significant importance in bacteria class distinction). Apparently, the signals between 1100 and 1700 cm^–1^ (part of the fingerprint region and amide I signal at ∼1600 cm^–1^) as well as between 2800 and 3000 cm^–1^ (aliphatic C–H stretching vibrations) are the most important for differentiating between the bacteria strains. However, one always expects to differentiate the two bacteria spectra to some extent in the calibration scores plot of the PLS-DA model. Hence, it is necessary to validate the actual ability of the PLS-DA model to distinguish the bacteria from each other by applying it to an independent dataset.

**Figure 5:**
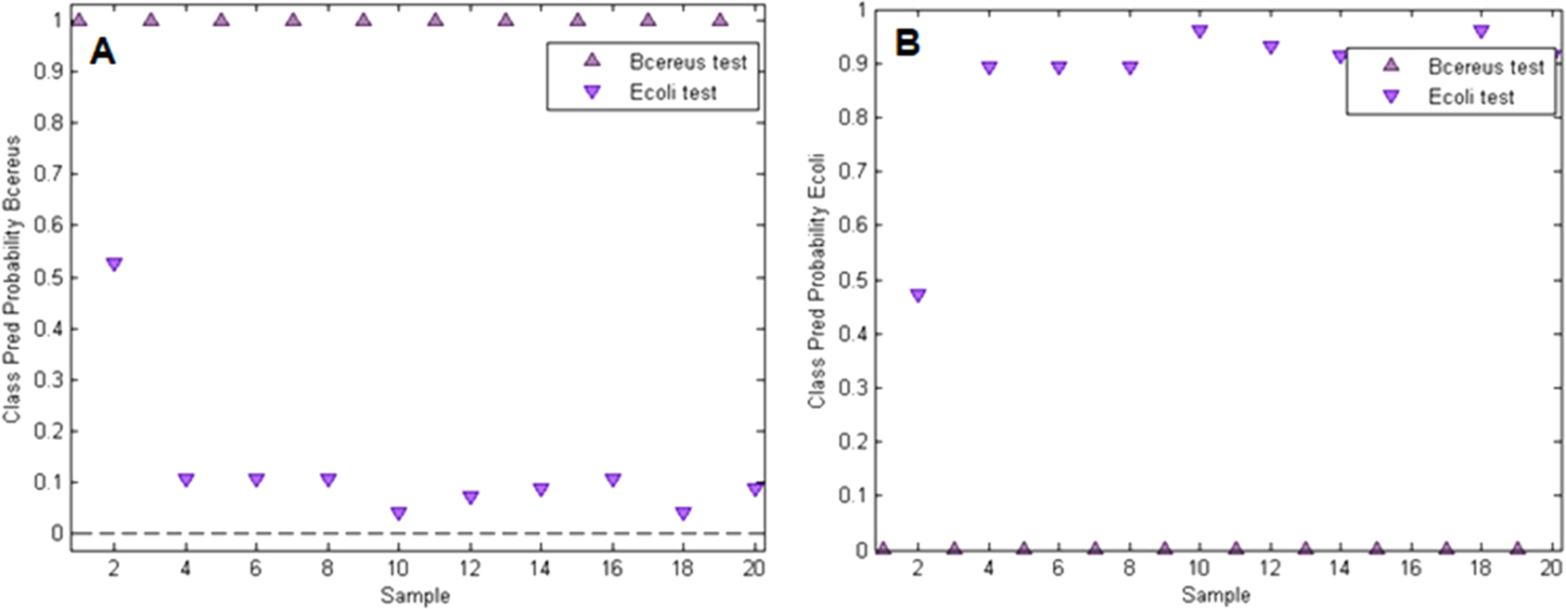
Validation results for the generated PLS-DA model; A: class prediction probability of *B. cereus* is close to 1 for all *B. cereus* Raman spectra, but it is around or below 0.1 for all *E. coli* spectra except for sample 2; B: class prediction probability of *E. coli* is 0.9 or higher for all *E. coli* spectra except for sample 2, whereas it is zero for all *B. cereus* Raman spectra.

Figure 5A,B shows the results of the PLS-DA model validation. The model with 1 latent variable correctly classifies 95% of the validation spectra of *E. coli* and *B. cereus*. The only false class assignment is an *E. coli* spectrum (sample 2) that is associated with the *B. cereus* class. This demonstrates that we successfully developed a PLS-DA model that can be applied to differentiate those bacteria strains on the *E. coli*-MIP with the high accuracy needed for selectivity studies.

### Differentiating between *E. coli* XL1 and *E. coli* BL21 (DE3) on *E. coli*-Imprinted Poly(styrene-*co*-DVB)

**Figure 6:**
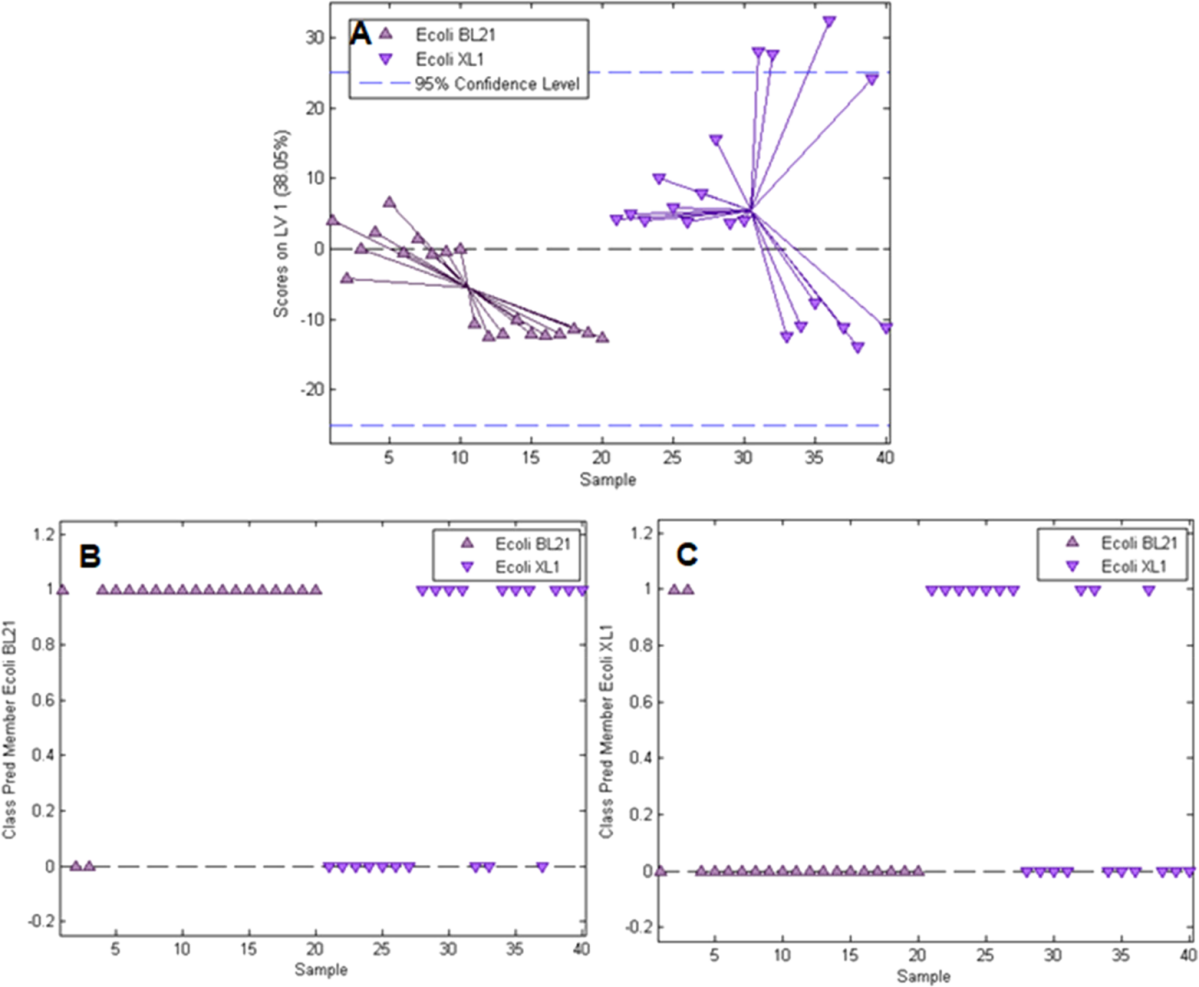
Calibration and validation results for the PLS-DA model distinguishing *E. coli* XL1 and *E. coli* BL21 (DE3). A: Scores plot for the model with 1 latent variable showing scores vs sample number, class prediction for *E. coli* BL21 (DE3) (B), and *E. coli* XL1 (C) (samples with a value of 1 are assigned to the respective class, whereas samples with a value of 0 are not).

To differentiate between the two *E. coli* strains XL1 (derivative strain of *E. coli* K-12) and BL21 (DE3) (derivative strain of *E. coli* B), two spots of each strain were applied to the *E. coli*-MIP, and 20 Raman spectra were acquired per spot. In contrast to the experimental setup when differentiating *E. coli* and *B. cereus* from one another, the PLS-DA model was calibrated using 10 spectra of each of the two spots per *E. coli* strain and applied to a validation dataset consisting of the residual (“unknown”) 10 Raman spectra of each of the spots. This was necessary because the two *E. coli* strains are expected to have more similar Raman spectra than *E. coli* (Gram-negative) and *B. cereus* (Gram-positive). Thus, when calibrating the PLS-DA model on a set of calibration spots and performing validation on a set of test spots, local differences in polymer composition might overpower the actual spectral differences between the two *E. coli* strains and thus make it impossible to identify the bacteria accurately. Setting up the model on both spots for each bacterium and using the “unknown” spectra from all of those spots for model validation should at least partly eliminate the impact of differences in polymer composition on the PLS-DA calibration. Figure 6A shows the scores plot resulting from model calibration.

As expected, distinguishing between two *E. coli* strains is not as straightforward as for two different bacteria species such as *E. coli* and *B. cereus*. The scores plot does show some overlap between *E. coli* XL1 and *E. coli* BL21 on the first latent variable. From the class predictions for *E. coli* BL21 (DE3) (Figure 6B) and *E. coli* XL1 (Figure 6C), one can see that there is quite a high number of false class assignments compared to the results for the *E. coli*/*B. cereus* model: 70% of Raman spectra are correctly classified (18 out of 20 for *E. coli* BL21 (DE3) and 10 out of 20 for *E. coli* XL1) compared to 95%. This observation is the result of the high similarity between *E. coli* strains and thus not surprising. However, we expect that this problem could be tackled using established techniques for selectively enhancing signals from the bacteria surfaces because the surface compositions of distinct *E. coli* strains are different^43^ and one could thereby circumvent the influence of inhomogeneous polymer composition.

### Differentiating between *B. cereus* and *L. lactis* and Applying the Model to a Mixture of the Two Bacteria Species

**Figure 7:**
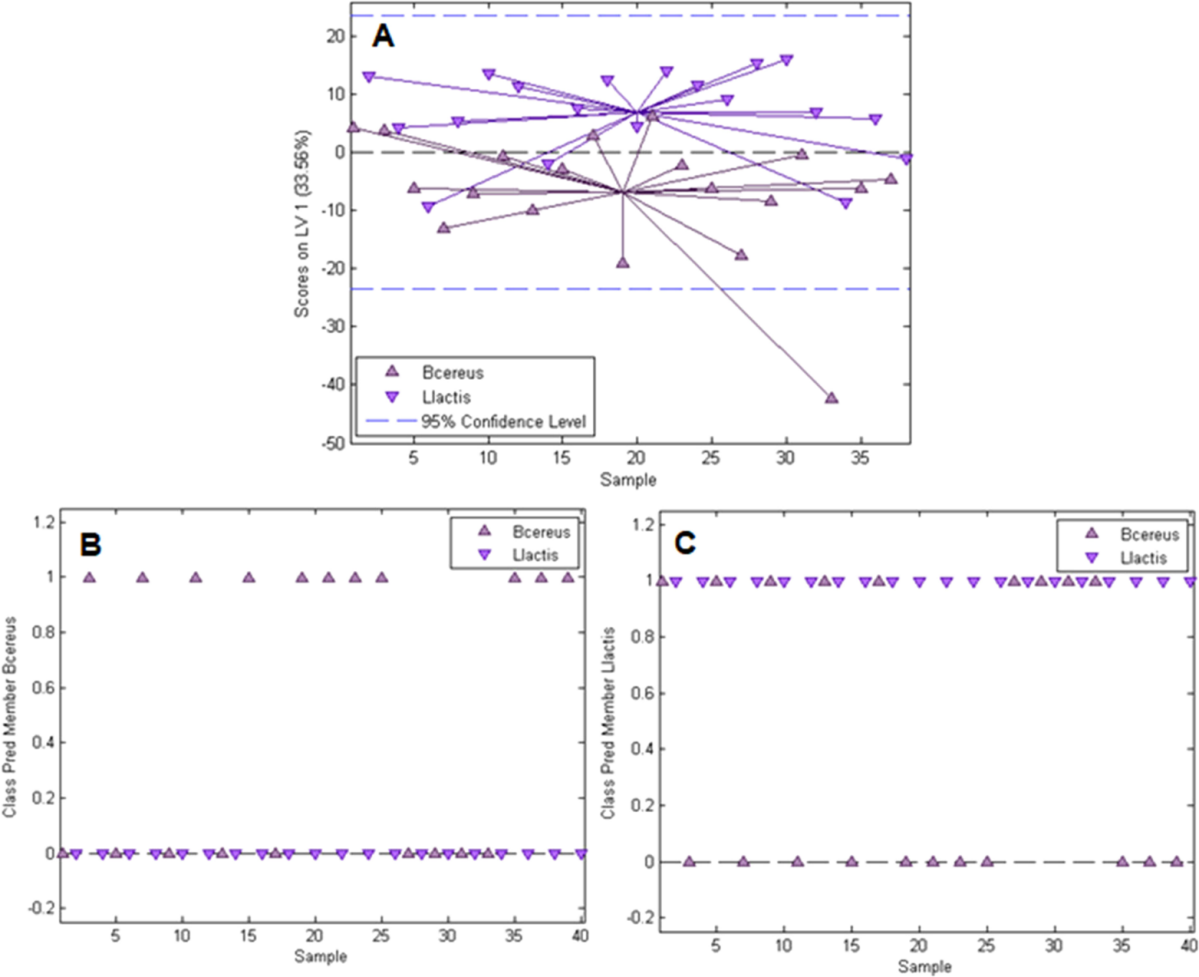
Calibration and validation results for the PLS-DA model for *B. cereus*/*L. lactis* differentiation and identification from a mixture. A: Scores plot showing scores on the first latent variable vs the sample; class prediction for *B. cereus* (B) and *L. lactis* (C) from the mixture (samples with a value of 1 are assigned to the respective class; samples with a value of 0 are not).

Applying Raman Microscopy-PLS-DA to assess the selectivity of *E. coli*-imprinted polymers requires the possibility to correctly identify different bacteria species from a mixture. This is especially the case when one desires to investigate selectivity of the MIP at conditions where several strains are competing for rebinding at once. Rod-shaped *B. cereus* and globular *L. lactis* are two species that are sufficiently different in shape to be distinguished visually. Thus, it is possible to determine the accuracy of their identification from a mixture, which makes them the most useful choice for this experiment. The PLS-DA model for differentiation between *L. lactis* and *B. cereus* was calibrated on two calibration spots on *E. coli*-imprinted poly(styrene-*co*-DVB) and applied to a spot containing a mixture of the two species. Figure 7A shows the scores plot obtained from the model calibration using one latent variable.

As one can observe from the scores plot, the distinction between *B. cereus* and *L. lactis* is a bit more challenging than for *E. coli* and *B. cereus*, which results from the fact that *B. cereus* and *L. lactis* are both Gram-positive bacteria, whereas *E. coli* is Gram-negative. The validation results shown in Figure 7B,C show that 11 out of 20 *B. cereus* spectra and 20 out of 20 *L. lactis* spectra are correctly identified in the mixture, adding up to a total of 31 out of 40 spectra accurately classified (77.5%). Similar to the differentiation of two *E. coli* strains, the use of techniques to selectively enhance bacteria signals compared to the polymer substrate will benefit the distinction and make more accurate the bacteria identification from mixtures possible.

### Differentiating between *E. coli*, *B. cereus*, and *L. lactis* on *E. coli*-Imprinted Poly(styrene-*co*-DVB)

**Figure 8:**
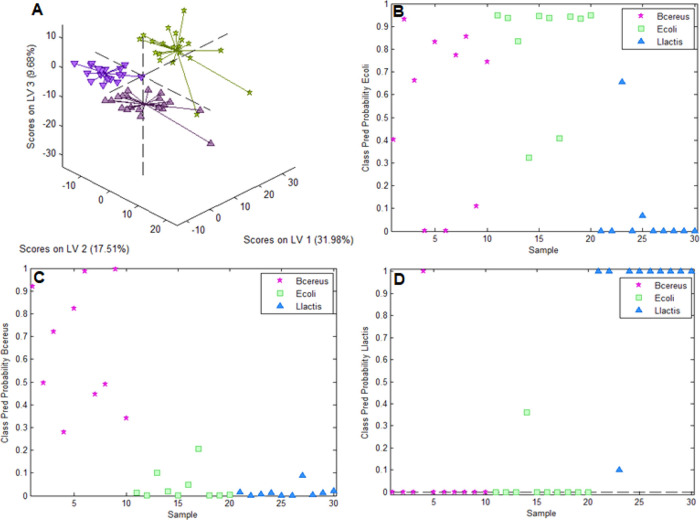
A: Scores plot of the PLS-DA model differentiating *E. coli*, *B. cereus*, and *L. lactis* on *E. coli*-imprinted poly(styrene-*co*-DVB) showing distinct clusters for *E. coli*, *B. cereus*, and *L. lactis*; B, C, and D: class prediction probabilities of *E. coli*, *B. cereus*, and *L. lactis* for the validation dataset.

Figure 8A shows the distinct clusters of the spectra of *E. coli*, *B. cereus*, and *L. lactis* in a scores plot resulting from the PLS-DA model with 3 latent variables. Experiments aiming at distinguishing more than two bacteria strains on *E. coli*-imprinted poly(styrene-*co*-DVB) were carried out in the same manner as for the 2 bacteria model (see the Experimental Section).

Figure 8D–B shows the class prediction probabilities for the *E. coli*, *B. cereus*, and *L. lactis* classes, respectively, resulting from the model validation. For *E. coli* (Figure 6B), 8 of 10 spectra are associated with the correct class. However, 6 *B. cereus* samples and one *L. lactis* spectrum are also classified as *E. coli*. In *B. cereus* (Figure 6C), only 5 of the 10 spectra have a class prediction probability of above 0.5 for the *B. cereus* class. For *L. lactis* (Figure 6D), all but one Raman spectrum are correctly classified, although one of the spectra belonging to the *B. cereus* class is erroneously associated with *L. lactis*. Comparing the validation results of the 3 bacteria strain model to the one using only 2 bacteria types indicates that adding a third class to the dataset substantially reduces the accuracy of the generated PLS-DA model (73% of spectra correctly classified compared to 95%). Difficulties in bacteria distinction beyond 2 different species on MIPs can be attributed to the fact that class differentiation relies on very small spectral differences, because most of the Raman signal intensity observed originates from *E. coli*-imprinted poly(styrene-*co*-DVB). In future experiments, tip-enhanced Raman scattering could serve to enhance bacteria signals exclusively without increasing the intensity of the polymer background spectrum, which may help in solving this problem.

## Conclusions

In this paper, we have presented the successful development of a novel method for the assessment of MIP selectivity based on Confocal Raman Microscopy and PLS-DA. Confocal Raman Microscopy provided a possibility to distinguish between imprints, polymer, and bacteria on the *E. coli*-MIP based on different signal intensities in their Raman spectra at 2908 cm^–1^. PLS-DA proved to be a powerful tool to differentiate *E. coli* and *B. cereus* based on their Raman spectra on *E. coli*-imprinted poly(styrene-*co*-DVB) with 95% of spectra correctly classified despite strong interfering Raman signals originating from the substrate. Distinguishing two different strains of *E. coli* or 3 different bacteria strains as well as applying the model to a mixture of bacteria on the *E. coli*-MIP is more challenging, which manifests in a reduced percentage (70%, 73%, and 77.5%) of the correctly classified spectra. Hence, in the future, the focus needs to be on selectively enhancing the bacteria signal to diminish interfering Raman bands from the polymer.

## Supporting Information Available

Detailed description of software features used for extraction of the *E. coli* spectrum on *E. coli*-MIP and procedures for calculation of the average spectra (PDF)

## Abbreviations

AFM: atomic force microscopy
MIP: molecularly imprinted polymer
PLS-DA: partial least squares discriminant analysis
QCM: quartz crystal microbalance
