# Supplementary material for: Raman Studies on Surface-Imprinted Polymers to Distinguish the Polymer Surface, Imprints, and Different Bacteria
Source: ACS Appl Bio Mater. 2021 Dec 23;5(1):160–71. doi: 10.1021/acsabm.1c01020 (PMC8767538; doi:10.1021/acsabm.1c01020)
Supplement: Supplementary file 1 — mt1c01020_si_001.pdf [file mt1c01020_si_001.pdf]

Supporting Information

Raman Studies on Surface-Imprinted Polymers to Distinguish Polymer Surface, Imprints, and Different Bacteria

*Birgit Bräuer, Felix Thier, Marius Bittermann, Dieter Baurecht, Peter A. Lieberzeit**

University of Vienna, Faculty for Chemistry, Institute of Physical Chemistry, Waehringer Strasse 42, 1090 Vienna, Austria

*E-mail: peter.lieberzeit@univie.ac.at

EXPERIMENTAL SECTION

Influence of topography on differentiating E.coli-imprints and polymer in poly(styrene-co-DVB)

Masks containing 427 spectra were generated for both *E.coli*-imprints and surrounding polymer using a threshold of 55.9 CCD cts for the polymer and 39.5 CCD cts for imprints. The corresponding average spectra were calculated using the Advanced Graph Average feature in the WITec Project FIVE software

Extraction of E.coli spectrum from spectra acquired on E.coli-imprinted poly(styrene-co-DVB)

When performing a Raman image scan of the *E.coli*-MIPs exposed to their template species in an area where bacteria were present on the MIP, the intensity of the poly(styrene-co-DVB) Raman spectrum overpowers the Raman signals of the bacteria. Thus, after background subtraction and cosmic ray removal, the True Component Analysis function of the Project FIVE software was used to demix the two components in the Raman image scan: manually created masks for "pure" poly(styrene-co-DVB) and *E.coli* on top of poly(styrene-co-DVB), respectively, help to define which image pixels belong to which component. Average spectra of each component were created and weighted "demixing" of the 2 components was finally performed to subtract the "poly(styrene-co-DVB") component from the "*E.coli* on poly(styrene-co-DVB)" component.

Differentiating between B.cereus and E.coli on E.coli-imprinted poly(styrene-co-DVB)

Average spectra of *E.coli* and *B.cereus* on *E.coli*-imprinted poly(styrene-co-DVB), respectively, were generated as follows: 20 spectra were acquired for each bacteria species (a spectral autofocus between 2783 – 3008cm^{-1} was performed prior to the acquisition of each spectrum), background subtraction and cosmic ray removal were performed as described in the experimental section, before the spectra were averaged using the average function in the WITec project FIVE software.
